# FDA- and EMA-Approved Tyrosine Kinase Inhibitors in Advanced *EGFR*-Mutated Non-Small Cell Lung Cancer: Safety, Tolerability, Plasma Concentration Monitoring, and Management

**DOI:** 10.3390/biom9110668

**Published:** 2019-10-30

**Authors:** Isabelle Solassol, Frédéric Pinguet, Xavier Quantin

**Affiliations:** 1 Unité de Recherche Translationnelle, Institut du Cancer de Montpellier (ICM), 34000 Montpellier, France; 2 Département de Pharmacie, Institut du Cancer de Montpellier (ICM), 34000 Montpellier, France; frederic.pinguet@icm.unicancer.fr; 3 Service d’Oncologie Médicale, Institut du Cancer de Montpellier (ICM), IRCM, INSERM, Univ. Montpellier, 34000 Montpellier, France; xavier.quantin@icm.unicancer.fr

**Keywords:** TKIs, NSCLC, side effects, TDM, quantification

## Abstract

Non-small-cell lung cancer (NSCLC) is the most common form of primary lung cancer. The discovery of several oncogenic driver mutations in patients with NSCLC has allowed the development of personalized treatments based on these specific molecular alterations, in particular in the tyrosine kinase (TK) domain of the epidermal growth factor receptor (*EGFR)* gene. Gefitinib, erlotinib, afatinib, and osimertinib are TK inhibitors (TKIs) that specifically target *EGFR* and are currently approved by the Food and Drug Administration (FDA) and the European Medicines Agency (EMA) as first line treatment for sensitive *EGFR*-mutant patients. However, these four drugs are associated with severe adverse events (AEs) that can significantly impact patient health-related quality of life and patient monitoring. EGFR-TKIs are commonly used together with other types of medication that can substantially interact. Here, we review approaches used for the management of TKI-AEs in patients with advanced NSCLC to promote the benefits of treatments and minimize the risk of TKI treatment discontinuation. We also consider potential TKI–drug interactions and discuss the usefulness of plasma concentration monitoring TKIs based on chromatographic and mass spectrometry approaches to guide clinical decision-making. Adjusting the most appropriate therapeutic strategies and drug doses may improve the performance therapy and prognosis of patients with advanced *EGFR*-mutated NSCLC.

## 1. Introduction

Non-small-cell lung cancer (NSCLC) accounts for more than 80% of lung cancer cases, with the majority of patients presenting with advanced disease at the time of diagnosis [1]. Advances in targeted and individualized treatment of NSCLC since the late 2000s have led to the development of epidermal growth factor receptor (*EGFR*)-tyrosine kinase inhibitors (TKIs), such as first-generation reversible gefitinib (Iressa) [2] and erlotinib (Tarceva) [3], and second-generation irreversible afatinib (Giotrif) [4]. These U.S. Food and Drug Administration (FDA)-approved drugs exhibit higher efficacy in patients harboring specific activating molecular alterations in the tyrosine kinase domain of *EGFR* (exons 18–21). Among them, the most common *EGFR*-activating mutations are exon 19 deletions (del19) (45%) and the L858R exon 21 substitution (40–45%) [5]. Exon 18 mutations occur much less frequently, accounting for approximately 5% of *EGFR* mutations.

Unfortunately, the response to first- and second-generation EGFR-TKIs is severely impaired in almost all patients by the unavoidable emergence of resistance to targeted therapy within 10–12 months. Several mechanisms of acquired resistance have been identified, but the secondary missense T790M mutation in *cis* with a primary activating mutant *EGFR* allele is over-represented and has been reported in more than half of all cases [6]. The T790M mutation is a gatekeeper mutation located in the ATP-binding pocket that increases the affinity of EGFR for ATP. However, besides first-generation EGFR-TKIs, second-generation EGFR-TKIs have also failed to overcome T790M-mediated resistance because the concentrations at which these drugs overcome T790M activity preclinically are too toxic and cannot be achieved in patients [7,8].

A third generation of irreversible EGFR-TKIs, such as osimertinib (Tagrisso) [9], has been developed to overcome acquired EGFR-TKI resistance due to *EGFR*-T790M mutation. The development of this treatment strategy, in contrast to first- and second-generation EGFR-TKIs that act on the central nervous system, has markedly improved both the clinical management and outcome of patients with advanced NSCLC [10]. Osimertinib has been demonstrated to be more efficient than conventional EGFR-TKIs and has been rapidly approved as a first-line treatment of advanced *EGFR*-mutated NSCLC [11]. Unfortunately, these compounds are also associated with the development of tumoral clone resistance after 6–17 months of therapy [12].

Most patients treated with EGFR-TKIs develop relatively controlled toxicity. However, only a small proportion discontinue EGFR-TKI therapy due to the appearance of significant and disabling adverse events (AEs) that impact quality of life [13,14]. These effects include dermatological reactions, diarrhea, hepatotoxicity, stomatitis, interstitial lung disease, and ocular toxicity [15]. Switching EGFR-TKIs, reducing doses, or discontinuing EGFR-TKIs temporarily before the patient develops severe AEs, notably hepatotoxicity, improves the performance of the drug and prognosis of patients with positive *EGFR*-mutated tumors [16]. Clinical practice can also involve the implementation of appropriate supportive care measures. To avoid AEs and control interindividual pharmacokinetic variations, drug doses should ideally be adapted for each patient, not according to body surface area as usual for chemotherapy, but rather according to blood concentrations of the drug. Therapeutic drug monitoring (TDM), based on the blood dosage of a drug, is a useful tool for providing individual treatment adapted by dose adjustment. In this review, we focus on the pharmacokinetic (PK) data and known drug interactions for each of the currently available EGFR-TKIs. We summarize the most severe adverse effects observed with first-, second-, and third-generation EGFR-TKIs. We provide an overview of analytical methods used for TDM and recent clinical trials on exposure response relationships of these novel agents. Finally, we discuss the role of oncologists in safely managing EGFR-TKIs use to ensure maximum patient benefit.

## 2. Currently Approved EGFR-TKIs

### 2.1. Erlotinib 

Erlotinib is a low molecular weight, reversible, and oral competitive inhibitor of EGFR tyrosine kinase that inhibits intracellular phosphorylation of EGFR. In *EGFR* mutation-positive tumors, erlotinib binds tightly to the ATP-binding site in the mutated kinase domain, which results in potent blocking of the MAP-kinase signaling. The blocking of signaling results in the interruption of tumoral cell proliferation and the activation of the intrinsic apoptosis pathway [17].

Erlotinib was the first EGFR-TKI assessed in the first-line treatment of locally advanced or metastatic NSCLC patients with *EGFR* activating mutations, and compared to standard chemotherapy [17]. Based on the positive results of the multicentric EURTAC trial, erlotinib was approved in 2013 for first-line therapy in advanced NSCLC in patients with del19 or the L858R substitution [18]. Erlotinib possesses several specific pharmacokinetic parameters as detailed in Table 1. If simultaneously administered with food, erlotinib must be administered during fasting because its absorption can be thus modified, delaying gastric emptying and its bioavailability [17]. The recommended dose of erlotinib is 150 mg daily, which can be administered either one hour before a meal (complete fasting) or two hours after a meal [19,20]. Erlotinib is rapidly absorbed and has a poor bioavailability and a long half-life (>36 h) [21]. Erlotinib is mainly metabolized by CYP3A4 in the liver. Erlotinib is also, but less extensively, metabolized by the CYP1A2, CYP1A1, and CYP1B1 enzymes through demethylation of side chains [22,23].

Since patients undergoing cancer treatment frequently use acid-reducing agents (ARAs), such as proton pump inhibitors (PPIs), H2-receptor antagonists (H2RAs), and antacids for palliation of dyspepsia, gastroesophageal reflux disease, or gastritis, erlotinib PK exposure has been widely assessed. The erlotinib PK values are significantly reduced upon concomitant administration with omeprazole, a PPI, and ranitidine, an H2RA, but not when administered with a staggered dosing approach to ranitidine. Therefore, concomitant use of erlotinib with PPI or H2RAs is not recommended in clinical practice. If treatment with an H2RA is required, erlotinib should be administered 10 h after the H2RA dosing and at least two hours before the next antagonist drug administration [24]. Kletzl et al. reported, in a retrospective study of 157 *EGFR*-mutated advanced NSCLC patients treated with erlotinib or gefitinib, that co-administration of acid-suppressant therapy did not significantly affect the efficacy (overall response rate (ORR) and progression-free survival (PFS)) in the overall cohort [24]. Finally, gastroesophageal reflux disease should clinically managed with the prescription of sucralfate and the limitation of gastric irritants and acid-rich foods.

Erlotinib exposure may be affected by concomitant use of other drugs that act on CYP3A4/1A1/1A2/1B1 and may result in re-distribution and changes in intracellular/tumor cell drug concentrations (Table 2). Thus, erlotinib should be used with caution with CYP3A4 inducers and inhibitors such as antifungal (e.g., ketoconazole), antibiotic (e.g., rifampicin), and antiretroviral drugs with an adaptation of the standard dose [25,26,27].

Finally, cigarette smoking has been shown to accelerate erlotinib catabolism, which is particularly important in the population of lung cancer patients. The plasma levels of erlotinib measured in current smokers almost doubled compared with those of former smokers and non-smokers 24 h after dosing [28]. Hamilton et al. compared the pharmacokinetic variables of erlotinib in current smokers and reported that an increased dose of erlotinib may benefit these patients [29]. Subsequent studies in patients with NSCLC confirmed this observation [17,30]. Therefore, the most crucial intervention is smoking cessation. Otherwise, increasing the dose of erlotinib by 50 mg is recommended at two-week intervals up to a maximum of 300 mg per day for concomitantly cigarette smoking patients.

### 2.2. Gefitinib 

Gefitinib is a drug that inhibits EGFR tyrosine kinase by targeting the ATP cleft within EGFR to prevent EGFR autophosphorylation, resulting in the inhibition of downstream signaling pathways, cell stasis, and/or cell death [31,32]. Since 2009, gefitinib has been a treatment option for locally advanced or metastatic NSCLC with sensitizing mutations of *EGFR* across all lines of therapy [33]. In July 2015, the FDA approved gefitinib for the first-line treatment of patients with metastatic NSCLC whose tumors harbor *EGFR* mutations, specifically exon 19 deletions or exon 21 L858R substitution [34]. The recommended dose of gefitinib is 250 mg once per day. Its bioavailability is independent of dose and unaffected by food to any clinically significant extent. After oral administration, gefitinib undergoes rapid blood clearance and has an extensive distribution volume (Table 1) [35]. Although the absolute bioavailability of the recommended dosage of gefitinib in patients is about 50%, the plasma concentration profiles after oral administration have shown that once-daily oral administration of gefitinib is appropriate, with steady-state achieved on day 7 [36]. A higher dose of 500 mg per day has been shown to be more effective but with higher toxicity [37]. The acid dissociation constant of gefitinib is similar to that of erlotinib, at approximately 5.4 [38]. Therefore, its solubility is highly pH-dependent, with a high solubility in the acid range and a solubility significantly lower in near-neutral pH (Table 2) [39].

The significance of the clinical impact of the co-administration of PPIs and H2RAs on gefitinib therapy in NSCLC patients has been extensively studied. The results of the area under the concentration-time curve and the maximum plasma concentration of gefitinib declined to 60% and 30%, respectively, after pretreatment with a high dose of ranitidine [40]. Kumarakulasinghe et al. and Zenke et al. reported that the concomitant use of PPIs and H2RAs with gefitinib did not significantly impact ORR, PFS, or drug toxicity [41,42]. However, the concentration of gefitinib remained related to its efficacy and the toxicity of the drug [43,44].

Similar to erlotinib, gefitinib is mostly cleared by hepatic metabolism via cytochrome P450 (CYP3A4 and CYP3A5). Gefitinib is mainly excreted as metabolites in the feces with around 90% of the received dose. The average elimination half-life was 48 h after administration (50–700 mg/day) in patients with solid tumors [45]. No significant effects of age, sex, bodyweight, or race on the pharmacokinetics of gefitinib have been reported to date.

### 2.3. Afatinib 

Afatinib is the first irreversible oral blocker of the ErbB family. It inhibits the activity of EGFR-1 (ErbB1), EGFR-2 (HER2, ErbB2), and EGFR-4 (HER4, ErbB4), and the transphosphorylation of ErbB3 [22,46]. The U.S. FDA has approved Giotrif for the management of locally advanced or metastatic patients with non-small cell lung cancer with the following characteristics: *EGFR* del19 or L858R. The approval of afatinib was based on the demonstration of improved PFS in a multicenter, international, open-label, randomized trial, the Lux-Lung 3 trial. Two phase III studies were conducted: Lux-Lung 3 and Lux-Lung 6. The results of the two studies showed an improvement in PFS compared with standard of care chemotherapy, especially in patients with tumors with sensitive *EGFR* molecular alterations [47]. In the randomized phase IIb Lux-Lung 7 trial, patients who received afatinib had significantly improved PFS and an improved objective response rate compared with those who received gefitinib (*n* = 159) [48]. However, no improvement in overall survival (OS) was shown. A Phase I dose-escalation study was conducted to determine the safety and tolerability of afatinib and the maximum tolerated dose pharmacokinetics. The results show that at a continuous dose of 40 mg/day for 28 days, afatinib was well tolerated and had an acceptable safety profile at the maximum tolerated dose (Table 1) [49,50].

Age, smoking status, and hepatic function have no effect on afatinib exposure. However, patients with low body weight or renal insufficiency are within the range of variability of afatinib exposure. After oral administration, afatinib plasma concentrations are reached for two to five hours and decrease at least bi-exponentially. Unlike other first-generation TKIs, no drug interaction occurs with CYP inducers or CYP inhibitors and acid-suppressant therapy at the approved dose of 40 mg of afatinib (Table 2) [51,52]. However, potent inhibitors or inducers, like rifampicine, probably impact the pharmacokinetics of afatinib [53]. Finally, the recommended dose of afatinib is 40 mg orally once daily; however, the dose can be increased to a maximum of 50 mg/day or decreased to a minimum of 20 mg/day, depending on tolerability [17,18].

### 2.4. Osimertinib

Osimertinib is an orally available, potent, and irreversible inhibitor of *EGFR*-sensitizing and T790M-resistant molecular alteration [10,54,55]. Osimertinib has high selectivity for *EGFR*-T790M or *EGFR*-L858R compared with the wild form. It has demonstrated a high objective response rate in T790M-positive NSCLC patients who progressed on a first-generation EGFR-TKI and had therefore been classified as a breakthrough compound for fast-track development [8]. Today, Osimertinib is approved by the European Union and the United States as a first-line treatment of patients with *EGFR*-positive metastatic NSCLC and for patients with *EGFR* T790M-positive metastatic NSCLC after disease progression on an EGFR-TKI [56].

Pharmacokinetic studies of osimertinib have shown that it is slowly absorbed with an absolute bioavailability of 70% [57]. Osimertinib is orally dispensed at a dose of 80 mg once daily [54] and is quite widely distributed. Osimertinib has a low to moderate clearance for a half-life of approximately 50 to 60 h in healthy volunteers and approximately 48 h in patients with metastatic NSCLC (Table 1) [58].

Based on a clinical pharmacokinetic study, food does not alter the bioavailability of osimertinib to a clinically significant extent [59]. A study conducted in healthy volunteers who received 80 mg of osimertinib once daily, and whose gastric pH was elevated by administration of omeprazole for five days, did not show any impact of this treatment on serum osimertinib concentrations [59]. No changes in the pharmacokinetic profile of osimertinib based on patients’ age, sex, body weight, ethnicity, smoking status, or renal impairment have been reported [60,61]. No apparent differences in the safety of osimertinib were observed between patients with normal hepatic function and those with mild or moderate hepatic impairment [62].

Osimertinib exhibits two active metabolites (AZ7550 and AZ5104) that can be detected at approximately 10% of exposure [58]. Both metabolites appear slowly in plasma after exposure, with a median time of more than 24 h. Osimertinib, AZ5104, and AZ7550 are mainly metabolized by CYP3A4 and CYP3A5 through oxidation and dealkylation [63]. CYP, glutathione, and cysteinyl glycine only contribute minimally [64]. Vishwanathan et al. demonstrated that CYP3A4 inhibitors have no clinically significant impact on osimertinib exposure. However, the concomitant exposure of osimertinib with strong CYP3A inducers, such as rifampicin, can significantly decrease osimertinib activity [65]. Therefore, osimertinib has been proposed for concurrent administration with CYP3A inhibitors, but co-administration of strong CYP3A inducers should be avoided with osimertinib if possible (Table 2). If not, the dose should be increased to 160 mg/day. Finally, Harvey et al. assessed the impact of osimertinib on the PK of simvastatin, a sensitive CYP3A substrate, and rosuvastatin, a breast cancer resistance protein (BCRP) substrate, in patients with *EGFR* mutation-positive NSCLC following progression on an EGFR-TKI. The authors showed that osimertinib had a less than two-fold change inhibitory effect on rosuvastatin exposure and recommended caution when using osimertinib with sensitive BCRP substrates [66].

Recently, Schoenfeld, et al. reported that the sequential combination PD-(L)1 blockade, such as pembrolizumab or durvalumab, and osimertinib was associated with severe immune AEs including pneumonitis and colitis [67]. The authors observed on a cohort of more than 120 patients that AEs appeared were related to a specific drug interaction rather than therapeutic class interaction between osimertinib and PD-1 blockade. Interestingly, toxicity only occurred when anti-PD-(L)1 treatment preceded EGFR-TKIs. In addition, these AEs usually appeared within weeks of starting osimertinib therapy [67]. Although the mechanisms explaining this synergistic toxicity remain unclear, it is possible that EGFR-TKIs differentially improve antigen-specific cytotoxic T-lymphocyte recognition and killing of tumor cells, further validating EGFR inhibitors as immunomodulatory agents that enhance checkpoint blockade [68].

## 3. Tolerability Profile of TKIs: Safety and Side Effects

### 3.1. First- and Second-Generation Toxicity

First- and second-generation EGFR-TKIs are well tolerated. Although AEs are usually minimal, they significantly affect the well-being and quality of life of patients and are therefore often assessed during treatment compliance. TKI side effects are classified by grade according to the National Cancer Institute common terminology criteria for adverse events. TKIs prescription can continue with diarrhea, mucositis, rashes, and paronychia, if well managed. In contrast, severe side effects, such as ocular disorders, interstitial lung disease, severe diarrhea, or severe hepatotoxicity, require immediate discontinuation of treatment. The most common grade 3 or 4 adverse effects reported with these drugs are rash and diarrhea, and less than 10% of patients discontinue treatment because of AEs (Table 2) [13].

Diarrhea can result from local irritation of the intestine because TKIs are mainly excreted in the stool. The effect of the inhibition of cKit in cells regulating intestinal motility has been proposed [69], which is ranked from grade 1 to 5 according to the frequency and severity of the form. Grade 1 is defined as an increase of less than four stools per day from baseline, grade 2 as 4 to six stools per day from baseline, grade 3 as more than 7 seven stools per day from baseline or incontinence, grade 4 includes life-threatening consequences, and grade 5 is death. The incidence of diarrhea in EGFR-TKI phase III clinical trials has been shown to range from 30% to around 90% [70]. Once the other causes of diarrhea have been eliminated (e.g., medication such as laxatives or antibiotics, excess consumption of fiber or diary product, or radiation toxicity [70]), the management of diarrhea induced by EGFR-TKI treatment involves adequate hydration and prescription of anti-diarrheal treatments as usually prescribed for chemotherapy-induced diarrhea; with a low-fiber diet of starchy foods, carrots, and bananas; and avoiding raw fruit and vegetables, dairy products, coffee, and alcohol. Loperamide is an effective therapy to decrease intestinal motility useful for TKI-induced diarrhea. For more severe cases, diphenoxylate and atropine or octreotide has been proposed. TKI treatment is rarely interrupted.

Mucositis has rarely been observed with first-generation TKIs, unlike second-generation TKIs. In the IPASS trial, 0.2% of patients treated with gefitinib reported grade 3 mucositis/stomatitis [71], whereas 8.7% of patients treated with afatinib in the Lux-Lung 3 experienced grade 3 mucositis/stomatitis [72]. After an initial assessment of oral health before the start of therapy with an EGFR-TKI, the oral cavity should be evaluated by a health care professional periodically throughout treatment and at treatment completion [73]. Typically, proposed prevention approaches include light but regular brushing with a soft brush, flossing, and saline rinsing. Patients should avoid commercial mouthwashes as they often contain alcohol, as well as alcohol and tobacco products, which can exacerbate the situation [74]. The treatment recommendations of EGFR-TKI-associated mucositis have been proposed by expert consensus and the ESMO guideline [75]. Briefly, oral patients can use a foam swab or a piece of gauze, which are softer and less abrasive, in low mucositis grade. For mild stomatitis, a patient should perform oral care every two to three hours; for patients with moderate-to-severe symptoms, oral care should be performed every one to two hours. Pharmacologic management strategies can be proposed in grade 2 (250–350 mg of erythromycin per day). Treatment for grade 2 stomatitis can also include triamcinolone in dental paste, two to three times daily. For grade 3, clobetasol ointment is used instead of triamcinolone in dental paste, and the erythromycin dose is increased to 500 mg daily [76]. EGFR-TKIs are generally maintained for grades 1 and 2. They are temporarily stopped for grade 3 until stomatitis or mucositis improves to grade 2, after which it is resumed at 50% of the initial dose, then increased if symptoms do not worsen [77].

The most common toxicity of EGFR-targeted agents involves skin structures resulting, most of the time, in early toxicities, including follicular acneiform eruptions, also termed acne-like rashes or folliculitis. The pathogenesis of TKI-induced skin toxicity may involve inflammatory changes within the epidermis as a reaction to the inhibition of intracellular kinase signaling pathways, leading to the modification of proliferation of basal keratinocytes, proinflammatory cytokines, early infiltration of T lymphocytes, and secondary infections. Skin reaction occurs within one to three weeks after the start of treatment and gradually fades over time. Later toxicities, which are increasingly common with the increasing number of patients treated with the first line in the long-term, occur and correspond to changes in hair texture, sometimes even alopecia, cutaneous xerosis (or skin dryness), and periungual lesions (paronychia and pyogenic granulomas). A rash typically appears within two weeks after starting treatment on the face, shoulders, upper part of the back, or on the chest. The incidence of all grades of rash in phase III clinical trials varies from 37% to 78% [78]. This rash is often accompanied by unpleasant sensations, itching, or pain.

The management of folliculitis depends essentially on its severity, the symptoms it causes and its impact on patient quality of life. Therefore, the explanation of the care strategies and symptoms management to the patients is especially important. Tetracyclines can be prescribed as a first-line treatment when an EGFR-TKI is introduced. The management of these complications varies from one team to another. Therapeutic attitudes may include local care at the beginning of treatment or at the time of the rash. Creams and ointments must be applied in sufficient quantities. Exposure to the sun is not recommended. The benefits of cyclic antibiotic therapy are real, and if their effectiveness is insufficient, an adaptation of the treatment should be discussed. A bacteriological sample is taken in cases of possible superinfection, and the patient is referred to a specialized dermatological consultation if necessary. Dose reduction is essential for grade 3, and a grade 4 rash leads to treatment discontinuity.

Beyond the modifications of the matrix and nails, which become fragile, brittle, and whose growth slows down, paronychia and pyogenic granulomas are the nail complications most often observed under EGFR-TKIs. Their incidence varies according to the series. A total of 56% of patients developed skin AEs in the French study conducted by Osio et al. [79], where only 11% of patients were treated with afatinib and 4% with erlotinib in the Lux-Lung 8 study [80]. The incidence of paronychia and pyogenic granulomas increases with the duration of treatment. The management of paronychia is based on local care combined with measures to prevent or limit injuries. If the appearance of the lesion suggests infection, a bacteriological sample is taken. Finally, although the side effects of EGFR-TKIs are often unpleasant, efforts should be made to keep patients on cancer treatment.

### 3.2. Third-Generation Toxicity

Osimertinib has a good tolerability profile in patients with locally advanced or metastatic *EGFR* mutation-positive NSCLC. In the dose-escalation cohort of the phase 1 AURA trial, osimertinib exhibited an acceptable safety profile, and no dose-limiting toxicity was reported at any dose tested (ranging from 20–240 mg per day) [81]. In subsequent studies (AURA 2 extension cohort [82], AURA 2 trial [83], AURA 3 trial [10], FLAURA trial [84], and the ASTRIS study [85]), the most common AEs related to osimertinib were rash, diarrhea, nausea, dry skin, paronychia, and stomatitis. Of note, in AURA 1 and 2 trials, four cases of deadly interstitial lung disease occurred that were considered potentially related to osimertinib by the investigators. Fatal adverse events were also reported in the ASTRIS study for four patients in the osimertinib group due to respiratory failure, pneumonitis, and ischemic stroke. Treatment with osimertinib had an acceptable tolerability profile in patients with normal liver function or mild to moderate hepatic impairment and no obvious disparities in safety have been reported to date [62]. Finally, osimertinib is a relatively well-tolerated TKI that appears to have a better toxicity and tolerability profile than the first and second generations.

## 4. Applications for Routine EGFR-TKI Dosage 

The current reference analytical method for the measurement of plasmatic EGFR-TKI concentration is liquid chromatography coupled online to tandem mass spectrometry (LC-MS/MS), which combines the advantages of chromatography (high selectivity and separation efficiency) and those of MS (structural information and further increase in selectivity), providing sensitive, specific, and rapid detection of several analytes simultaneously in a single analytical run. Several bioanalytical assays based on LC-MS/MS for the determination of erlotinib and for OSI-420, the active metabolite of erlotinib, in plasma have been reported (Table 3) [19,86,87,88]. Lankheet, et al. applied this approach for the quantification of erlotinib and *O*-desmethyl erlotinib in plasma and lung tumor tissue samples with an low limit of quantification (LLOQ) of 5.0 ng/mL and 50 ng/g in plasma and tumor tissue, respectively, using 50 μL of sample [89]. The study of the long-term effect of erlotinib on CYP3A activity was recently assessed in 19 NSCLC patients by analyzing CYP3A activity before and after TKI treatment. The authors observed a two-fold induction in CYP3A activity in patients after two months of erlotinib treatment compared with the activity before the start of the treatment [90]. This result demonstrates the importance of proposing individualized treatment with erlotinib if patients are co-prescribed drugs metabolized by CYP3A during erlotinib treatment.

Gefetinib quantification has been also largely investigated using LC-MS/MS (Table 3) [88,98,105,106,107,108] (Jones, Stafford et al. 2002, Zhao, He et al. 2003, Guetens, Prenen et al. 2005, Chahbouni, den Burger et al. 2009, Bouchet, Chauzit et al. 2011, Wang, Lim et al. 2011). Gefitinib’s metabolites have been poorly investigated. Zheng et al. reported a sensitive, selective, and rapid method for the simultaneous quantification of gefitinib and its major metabolites, but only in mouse plasma [109]. The first validated assay for afatinib quantitation in EDTA plasma, with simultaneous determination of erlotinib and gefitinib, was reported in 2016. The authors developed a specific LC-MS/MS for human and mouse plasma [92,94]. More recently, the same group successfully validated a quantitative LC-MS/MS assay for encorafenib (a new generation B-Raf inhibitor), erlotinib, gefitinib, *O*-desmethyl-gefitinib and afatinib in human plasma sing a simple sample pre-treatment procedure and LC-MS/MS [110].

Although LC-MS/MS represents a robust and sensitive quantification technique, it has several shortcomings that have so far prevented its common use in clinical settings for patient monitoring. First, the high initial purchase cost of the instrument and the high maintenance costs are prohibitive for many hospital laboratories. Second, LC-MS/MS is a sophisticated technology that requires highly specialized scientific staff. Finally, the automation of LC-MS/MS systems remains difficult to achieve. Plasma sample preparation and extraction procedures are still conducted manually by laboratory staff, which is slow (usually at least four hours to prepare and analyze a sample) and laborious, especially in routine.

Alternative techniques using LC have been developed to address these concerns, in particular to minimize the analytical run time of gradient elution. Ultra-high-performance liquid chromatography (UHPLC), an ultra-high-pressure system, allows the use of columns with small particles and small diameters, has a faster rate of analysis, a lower solvent consumption (about half), and a positive effect on the efficiency and analysis time [111]. Thus, UHPLC-MS/MS has been assessed for TKI quantification in human plasma samples. Bouchet et al. reported a successful assay quantification of nine TKIs within four minutes of run time using a Waters Acquity-UPLC^®^ system with a BEH C18-50 × 2.1 mm column [105]. Van Erp et al. used an UHPLC Acquity BEH C18 analytical column (100 mm × 2.1 mm ID, 1.7 *μ*m particle size) coupled to an Acquity TQ tandem MS detector to rapidly quantify six TKIs (imatinib, sunitinib, nilotinib, dasatinib, pazopanib, and regorafenib) and two active metabolites (N-desmethyl imatinib and N-desethyl sunitinib) [112]. Huynh et al. described the analysis of 14 currently used TKIs with an Acquity UPLC BEH C18 1.7 mm (2.1 × 50 mm; pore size 1.9 mm) analytical column coupled to a tripled quadrupole with an analysis time of five minutes per run [113]. He et al. developed and validated a simple and sensitive UHPLC-MS/MS method for the simultaneous quantification of eight commercial TKIs including erlotinib [114]. Finally, Merienne et al. proposed the quantification of 17 TKIs and two metabolites in human plasma simultaneously, covering a very large panel of TKIs [95].

One of the main advantages of the HPLC–UV assay is its relative high availability in small hospitals laboratories. Since it is important to consider the costs and accessibility of analytical instruments and the possibility of clinicians monitoring TKI concentrations in plasma, this approach could be an alternative methodology. Thus, Faivre et al. were the first to successfully develop a simple, sensitive, and cost-effective HPLC–UV method to simultaneously quantify gefitinib and erlotinib in human plasma from NSCLC patients [115]. The LLOQs found in this study were 20 ng/mL and 80 ng/mL for gefitinib and erlotinib, respectively. Recently, Ni et al. successfully developed a hybrid quadrupole-Orbitrap MS (Q-Exactive) method for concurrent determination of several TKIs including erlotinib and gefitinib [116]. The method used in this study offers an attractive LLOQ of 0.05 ng/mL for erlotinib and gefitinib.

The determination of the concentration of osimertinib in biological samples has been evaluated only recently. Osimertinib could be measured alone or simultaneously with other TKIs by LC-MS/MS [93,103,104,117,118]. The results reported contrasting data on osimertinib’s stability in plasma. Dickenson et al. showed that osimertinib was very unstable, whereas Rood et al. argued that osimertinib remained stable for a few hours at room temperature [64,103]. This discrepancy needs to be further clarified, as the result could have a significant effect on patient sampling. Given the instability of osimertinib at room temperature in plasma and blood, Marijn-Veerman et al. recommend that to minimize osimertinib deprivation, blood and plasma samples should be stored and processed only in ice (at 0 °C) [101]. Otherwise, it is not possible to accurately measure osimertinib concentrations as part of therapeutic drug monitoring.

## 5. Conclusions and Perspectives 

EGFR-TKIs are currently the standard first-line treatment of patients with advanced NSCLC with activating *EGFR* mutations. These targeted therapies are usually well tolerated comparative to conventional chemotherapy. The self-oral administration of the drug positively contributes to improving patient compliance and reducing the use of hospital resources [119]. However, this non-invasive route of administration is also associated with unpredictable bioavailability, increased risk of drug interactions, and concerns about patient safety, surveillance, and follow-up. Chronic EGFR-TKIs underdosage may favor the selection of resistant clones and thus disease progression, and overdosage may increase the risk of dose-related side effects. Self-administration also means that the severity of side effects can be considerably impacted by patient behavior (e.g., dietary habits, self-medicating, smoker status). To date, EGFR-TKIs can be administered in advanced NSCLC for a long period of time, usually until the disease progresses or unacceptable toxicity occurs. As early intervention with supportive care strategies can minimize the intensity of adverse reactions or allow for appropriate dose adjustments without the need to discontinue treatment, acute toxicity follow-up and side effect management is of major interest.

Therefore, medical oncologists should not only understand the AEs associated with these drugs, but also develop the necessary skills to detect and treat them to reduce morbidity and mortality as well as premature discontinuation of treatment. Patient education on these strategies is also of major importance and can improve patient tolerance, quality of life, and overall treatment outcome by initiating supportive care soon after symptoms occur. Within this guidance and preventive strategy, the pharmacist can play a major role. Good knowledge of disease monitoring, response milestones, and the risks versus benefits of the treatment options allows pharmacists to better advise patients or counsel patients more effectively. Nurses and prescribers are qualified to monitor toxicity risks as closely as possible. Unfortunately, they do not necessarily do so at regular intervals. Patients can connect with pharmacists who can provide them additional information or complementary recommendations on AEs without the need for patients to rapidly contact prescribers. When more complex or serious AEs appear, pharmacists who work closely with nurses and prescribers can propose the most appropriate therapeutic strategies. Finally, medical oncologists able to understand AEs along with patient education and collaboration between different medical professional groups for therapeutic drug monitoring systems are needed, but further research on the aforementioned methods of relief for continued TKI use should be also performed in the future.

The importance of proactive patient follow-up must be emphasized rather than relying on self-reporting between clinical visits. A randomized trial showed that patients receiving chemotherapy for advanced cancer, web-based symptom reporting with automated clinician e-mail alerts resulted in better health-related quality of life, fewer emergency room visits, fewer hospitalizations, and superior quality-adjusted survival [120].

Recently, Cheema et al. assessed the impact of an interprofessional, proactive follow-up algorithm on the incidence of dose interruptions, reductions, and the severity of AEs in patients on afatinib. The authors reported that over half of first AEs and one third of all grade 1-2 and grade ≥3 AEs were detected through proactive follow-up. This multi-disciplinary AE algorithm resulted in a low rate of costly emergency room or urgent clinic visits and a reduced incidence of severe drug-related AEs and discontinuation therapy.

In conclusion, when closely monitoring patients, the ability of clinicians to make rapid therapeutic decisions in the event of medical complications is critical in the diagnosis and management of TKI-related side effects. This is even more decisive in the event of serious or even life-threatening complications. However, good clinician–patient communication, drug patient education, early detection and management of drug side effects, and monitoring of disease response with plasma concentration quantification can also improve patient outcomes. Close cooperation between oncologists, internists, pharmaceutics, and nurses should become the gold standard in the comprehensive clinical care of advanced NSCLC patients receiving TKIs.

## Figures and Tables

**Table 1:** Summary of steady-state pharmacokinetics of 4 TKI after multiple once daily oral doses.

Parameter and unit	Erlotinib	Gefetinib	Afatinib	Osimertinib
Usual starting dose (mg)	150	250	40	80
AUC_τ,ss_	27 (ng h/mL)	258 (ng h/mL)	631 (ng h/mL)	9570 (nmol h/L)
C_max,ss_	1521 (ng/mL)	101(ng/mL)	38.0 (ng/mL)	550.4 (nmol/L)
t_max,ss_ (h)	4	4	3	4
t_1/2_,_ss_ (h)	36	52	37	48.6
CL/F_ss_ (L/h)	4.5	46	1070	17.7
V_z_/F_ss_ (L)	232	1700	2870	1216
Protein binding (%)	95	90	95	NA

Abbreviations: AUC_τ_,ss area under the drug plasma concentration–time curve at steady state over a uniform dosing interval τ, CL/Fss clearance of drug from plasma after oral administration at steady state, Cmax,ss maximum drug concentration in plasma at steady state, ss steady state, t,ss terminal elimination half-life at steady state, tmax,ss time to reach Cmax,ss, Vz/Fss (apparent) volume of distribution at steady state.

**Table 2:** Oral agents for treatment and significant interactions of metastatic lung cancer.

TKIs	Recommended Dosage/Day	Indication	Common AEs	Drug-Drug Interactions
Erlotinib	150 mg 1 h before or 2 h after food	first-line for advanced NSCLC	rash, diarrhea, edema, cough, conjunctivitis	inducer of CYP1A2 inhibitor of CYP3A4, CYP1A1 and CYP2C8, medication that alter gastric pH
Gefitinib	250 mg +/− food	first-line for advanced NSCLC	skin reaction, rash, anorexia, stomatitis diarrhea, paronychia	inducers of CYP3A4, and CYP2D6, P-gp, inhibitors of CYP2C19 and CYP2D6, UGT1A1, medication that alter gastric pH
Afatinib	40 mg 1 h before or 2 h after food	second-line for advanced NSCLC	eruption rash dry ski, diarrhea, loss of appetite, stomatitis	negligible metabolism via CYP pathways; substrate and potential inhibitor of P-gp
Osimertinib	80 mg +/− food	third-line for advanced NSCLC	diarrhea, rash, dry skin, nail toxicity, ILD, QTc prolongation, ocular desorder cardiomyopathy	inducers of CYP3A

Abbreviations: QTc, QT interval corrected for heart rate, ILD Interstitial lung disease, CYP cytochrome P450, UGT, uridine diphosphate-glucuronosyltransferase, P-gp P-glycoprotein, +/− food with or without food, AEs adverse events, NSCLC non-small cell lung cancer.

**Table 3:** LC-MS/MS methods for the quantification of the 4 TKI in human plasma.

Generation Molecule	Analyte	IS	Parent Ion (m/z)	Product Ion (m/z)	Column	Calibration Range (ng/mL) or (nM)	LOQ (ng/mL) or (nM)	Extraction	Reference
1^st^	Erlotinib	D8-imatinib	394.2	277.9	C18 (100 × 2.1mm, 3µm)	50–3500	50	LLE	[91]
		Elotinib-^13^C_6_	394.1	278.1	C18 (30 × 2.1mm, 1.7µm)	5–4000	5	PP	[92]
		Erlotinib- ^2^H_6_	394.2	278.0	C18 (50 × 2.1mm, 2.6µm)	20–4000	20	LLE	[93]
		Erlotinib-d6	395.2	279.2	C18 (100 × 2.1 mm, 1.7µm)	25–5000	25	PP	[94]
		Erlotinib-^13^C_6_	394.1	278.1	C18 (50 × 2.1mm, 1.6µm)	25–5000	25	SPE	[95]
		Omatinib mesylate	394.0	278.3	C18 (50 × 2.1mm, 3.5µm)	0.01–250 nM	0.01 nM	LLE	[96]
		Midazolan	394.2	278.0	C18 (150 × 4.6mm, 5µm)	10–5000	10	LLE	[87]
1^st^	Gefitinib	Vatalanib	447.6	128.2	C18 (50 × 2.1mm, 3.5µm)	0.5–1000	0.5	LLE	[97]
		Gefitinib-^2^H_8_	447.0	128.1	C18 (50 × 2.1mm, 1.6µm)	4–800	4	SPE	[95]
		Imatinib mesylate	447.0	128.3	C18 (50 × 2.1mm, 3.5µm)	0.01–100 nM	0.01 nM	LLE	[96]
		O-methyl gefitinib-d3	447.1	128.2	C18 (150 × 2.1mm, 5µm)	5–1000	5	PP	[98]
		Gefitinib-d8	447.1	128.05	C18 (30 × 2.1mm, 1.7µm)	2–1600	5	PP	[92]
2 ^nd^	Afatinib	Cyclobenzaprine	486.1	276.0	C18 (50 × 2.0mm, 3µm)	0.5–500	0.5	LLE	[99]
		Afatinib-^13^C_6_	486.2	370.9	C18 (50 × 2.1mm, 2.6µm)	5–250	5	LLE	[93]
		Cyclobenzaprine	486.1	371.0	C18 (50 × 2.0mm, 3µm)	5–500	4.3	LLE	[100]
		Afatinib-d6	486.0	112.0	C18 (50 × 2.1mm, 1.7µm)	1–100	1	LLE	[101]
		Afatinib-^13^C_6_	486.1	371.0	C18 (50 × 2.0 mm, 5µm)	2–200	2	LLE	[102]
		Imatinib mesylate	486.0	371.3	C18 (50 × 2.1mm, 3.5µm)	0.05–100 nM	0.005 nM	LLE	[96]
		Afatinib-^13^C_6_	486.0	305.1	C18 (50 × 2.1mm, 1.6µm)	4–800	4	SPE	[95]
3 ^rd^	Osimertinib	Ppazopanib	500.2	72.1	C18 (50 × 2.1 mm, 1.7 µm)	1–1000	1	LLE	[103]
		[13C,2H3]-osimertinib	500.1	72.1	C18 (50 × 2.1mm, 2.6µm)	5–1000	5	LLE	[93]
		Erlotinib-d6	500.5	72.5	C18 (50 × 2.1mm, 1.7µm)	10–1000	10	LLE	[101]
		IS-0741	500.4	385.3	C18 (50 × 2.1 mm, 1.7μm)	0.5–100	0.5	PP	[104,105,106]

Abbreviations: IS internal standard, LOQ Limits of quantification, LLE liquid-liquid extraction, SPE solid-phase extraction, PP protein extraction, LC-MS/MS liquid chromatography tandem mass spectrometry.
